# *Letter to the Editor:* Medical Cannabis in Canada: The Need for Insurance Coverage Expansion

**DOI:** 10.1089/can.2023.0120

**Published:** 2024-02-12

**Authors:** Stephanie Lunn

**Affiliations:** Medical Affairs, Aurora Cannabis, Comox, British Columbia, Canada.

**Dear Editor:**

In 2021, Canadians purchased over 16 million packages of cannabis for both medical and nonmedical purposes.^1^ However, while individuals may report using cannabis for medical purposes, it does not necessarily mean they are purchasing from medical channels. The number of people registered with federally licensed producers for medical cannabis has been consistently decreasing since the fall of 2020^2^ and data from Canada and the United States continues to show that people will access cannabis through recreational channels for medical purposes. The 2022 Health Canada cannabis use survey found 73% of respondents who reported using cannabis for medical purposes did not have an authorization document from a health care professional.^3^ This statistic has remained relatively static since 2020, indicating that the majority of Canadians who use cannabis for medical purposes continue to access cannabis through means other than the medical channel.^3^

It is not ideal for the public to use cannabis for medical purposes without medical oversight, and yet, 56% of Canadian respondents who reported using cannabis for medical purposes did so by purchasing their cannabis from the legal adult-use market; legal storefronts were the most common place of purchase (45%).^3^ This could be detrimental given there is evidence that cannabinoids, particularly cannabidiol,^4–7^ can result in significant drug–drug interactions and that individuals who seek out cannabis for medical purposes often have complex medical conditions. Additionally, patients miss out on the knowledge cannabis producers provide via various patient support teams, direct delivery and consistent product stock of more medically focused cannabis products, when they move to purchase their cannabis from the adult-use market.

One reason Canadians continue to seek out their cannabis for medical use from the adult-use market is the lack of widespread insurance coverage for medical cannabis. Though the scientific evidence supports the use of medical cannabis in a number of conditions, in Canada, medical cannabis is treated differently from other medications (i.e., it is not covered by insurance and is not always easily accessible by patients who could benefit from it).

While medical cannabis can be claimed on income tax returns, insurance coverage is both limited and variable in Canada; 91% of surveyed Canadians who use cannabis for medical purposes reporting they do not have insurance coverage for it.^3^ In most cases, while many group insurers do offer medical cannabis programs, it is up to employers to include such coverage. When covered, claims are typically subjective to restrictions on medical conditions for which medical cannabis can be used for along with yearly monetary caps and at times, administrative procedures are required to prove all other pharmaceutical options have been attempted.^8^

The lack of insurance coverage is compounded by the taxation of medical cannabis via an excise tax; the Arthritis Society reported that the sales and excise taxes on medical cannabis cost an average arthritis patient almost $2,000/year, based on average dosage.^9^ With monthly costs of medical cannabis reaching as high as $500 a month,^10^ the absence of insurance coverage puts a significant economic burden on many patients. These financial issues are likely why many source their cannabis from the adult-use market, which has continued to experience significant price compressions as a result of steep competition.^11^

A solution to these financial barriers could be to offer insurance coverage for medical cannabis. This would offset the costs for patients who have been authorized for medical cannabis and aid in legitimizing medical cannabis to the public and health care practitioners. Such coverage already exists in Canada, with Veterans Affairs Canada's (VAC's) reimbursement policy for medical cannabis. VAC's policy was in response to research and the department's panel of medical experts and could serve as a template to expand coverage in other public drug plans and/or any future national pharmacare programs.

Additionally, given the significant amount of evidence showing use of cannabis for medical purposes can lead to a reduction in the usage of other more harmful pharmaceuticals, like opioids, expanding insurance coverage for medical cannabis has the potential to meaningfully combat the opioid crisis in Canada.^12–15^ There have been 32,632 apparent opioid toxicity deaths between 2016 and 2022 in Canada, and the crisis continues to worsen year after year.^16^ In the period of January–June 2022, the were about 20 deaths per day caused by apparent opioid toxicity, while in 2016, there were about 8 deaths per day for the same time period.^16^

With the above actions taken, those seeking to use cannabis for medical purposes can do so safely under the guidance of their preferred health care practitioner without experiencing financial hardships.

## Abbreviation Used

VAC's: Veterans Affairs Canada's
